# Conformational analysis, stereoelectronic interactions and NMR properties of 2-fluorobicyclo[2.2.1]heptan-7-ols

**DOI:** 10.3762/bjoc.8.137

**Published:** 2012-08-02

**Authors:** Fátima M P de Rezende, Marilua A Moreira, Rodrigo A Cormanich, Matheus P Freitas

**Affiliations:** 1 Department of Chemistry, Federal University of Lavras, P.O. Box 3037, 37200-000, Lavras, MG, Brazil; 2 Chemistry Institute, State University of Campinas, P.O. Box 6154, 13083-970, Campinas, SP, Brazil

**Keywords:** conformational analysis, 2-fluorobicyclo[2.2.1]heptan-7-ols, hydrogen bond, ^1h^*J*_F,H(O)_ coupling constant, quantum-chemical calculations

## Abstract

Four diastereoisomers of 2-fluorobicyclo[2.2.1]heptan-7-ols were computationally investigated by using quantum-chemical calculations, and their relative energies were analyzed on the basis of stereoelectronic interactions, particularly the presence or otherwise of the F∙∙∙HO intramolecular hydrogen bond in the *syn*-*exo* isomer. It was found through NBO and AIM analyses that such an interaction contributes to structural stabilization and that the ^1h^*J*_F,H(O)_ coupling constant in the *syn*-*exo* isomer is modulated by the *n*_F_→σ*_OH_ interaction, i.e., the quantum nature of the F∙∙∙HO hydrogen bond.

## Introduction

**Figure 1:**
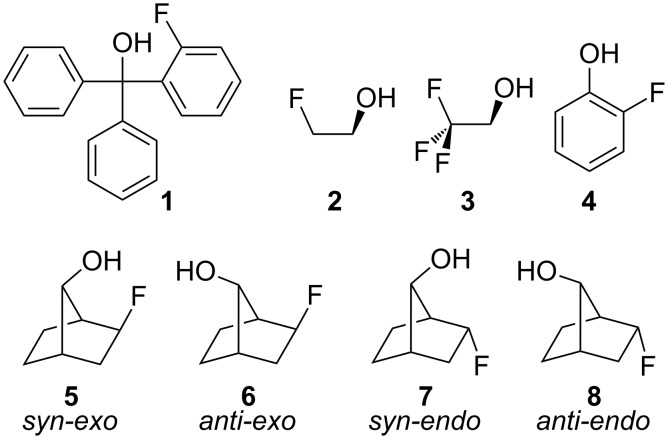
Some organofluorine compounds and the 2-fluorobicyclo[2.2.1]heptan-7-ols (**5**–**8**) theoretically studied in this work.

Intra- and intermolecular hydrogen bonds (HB) play an important role in determining the molecular arrangements and properties, as well as reactivity of a wide range of chemical and biological systems [1]. However, it has been argued that organic fluorine hardly ever participates in HB, due to the poor proton acceptor ability of the fluorine atom [2]; nevertheless, there are some instances of organofluorine compounds forming seven-membered hydrogen bonds [3], while the absence of HB in some monocyclic fluorohydrins has been shown to be due to geometric restrictions imposed by the ring size [4]. While structure **1** in Figure 1 exhibits a F∙∙∙HO intramolecular HB, structures **2**, **3** and **4** do not experience such an interaction [5–8]. However, 2-fluorophenol (**4**) shows a through-space (TS) coupling constant ^1TS^*J*_F,H(O)_ of ca. 5 Hz, which has been ascribed as being due to the overlap of electronic clouds between F and hydroxy H rather than to hydrogen bonding [8], while the corresponding SSCC in 8-fluoro-4-methyl-1-naphthol, which exhibits F∙∙∙HO intramolecular HB, is substantially higher, i.e., (−)28.4 Hz [9].

In fact, the importance of NMR scalar spin–spin coupling constants (SSCCs) transmitted through hydrogen bonds emerged, fundamentally, from the observation of ^1h^*J*_15N,H_ and ^2h^*J*_15N,15N_ SSCCs for DNA and RNA molecular systems [10–11]. ^19^F is a suitable nucleus for NMR analysis, since it has spin 1/2, and thus, F∙∙∙HO intra/intermolecular HB in biological systems can be readily assessed through *J*_F,H(O)_ SSCCs. This seems relevant because the replacement of a hydrogen by a fluorine atom in a molecule does not have a significant steric effect, but it suppresses adventitious metabolism, influences the pKa of functional groups, and alters solution conformation [12].

In this context, conformational screening and theoretical evaluation of 2-fluorobicyclo[2.2.1]heptan-7-ols (2-fluoronorbornan-7-ols, compounds **5**–**8** in Figure 1) represents an adequate approach to rationalize the role of F∙∙∙HO intra/intermolecular HB, since these model compounds are less flexible (easier to analyze) than others based on, e.g., the 2-fluoroethanol fragment, and allow the energetic comparison with a pool of diastereoisomers that do not experience such an interaction. Intramolecular interactions between vicinal F and OH groups have already been investigated in cyclic compounds (including aromatic rings) [4], and the present study extends such analysis to aliphatic compounds capable of forming six-membered rings through hydrogen bonding.

## Results and Discussion

**Figure 2:**
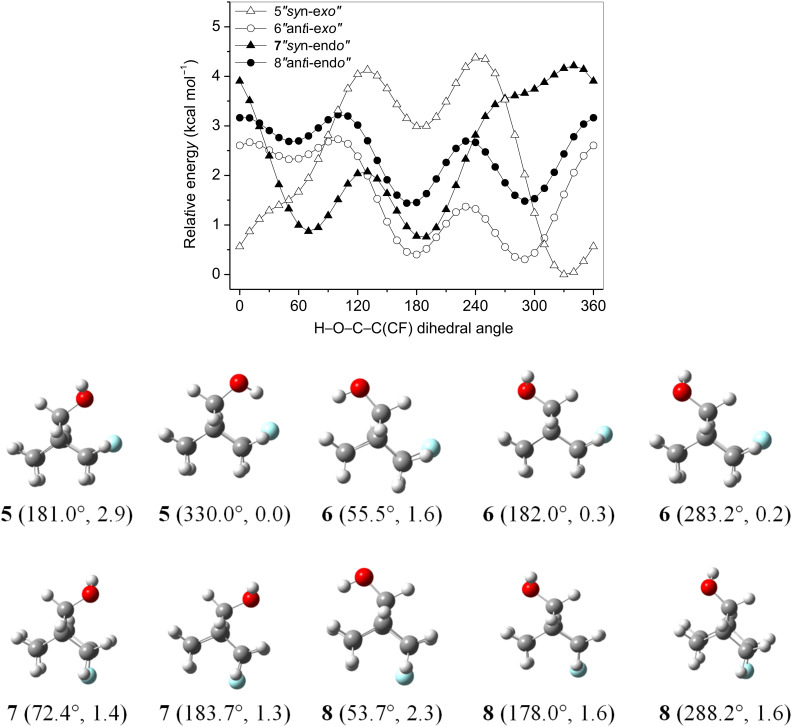
Potential energy surfaces for the diastereoisomers of 2-fluorobicyclo[2.2.1]heptan-7-ols (**5**–**8**), obtained at the HF/6-31g(d,p) level, and the optimized minima at the MP2/aug-cc-pVDZ level (θ dihedral angles and relative energies, in kcal mol^−1^, in parenthesis).

The hydroxy group of the 2-fluorobicyclo[2.2.1]heptan-7-ols undergoes rotation, giving rise to the stable conformers (energy minima) of the potential energy surfaces (PES) in Figure 2, which were obtained by computing the relative energies of **5**–**8** upon scanning of the H–O–C–C(CF) dihedral angle (θ) in steps of 10° at the HF/6-31g(d,p) level, using the Gaussian09 package of programs [13]. Each minimum was subsequently optimized at the MP2/aug-cc-pVDZ level, and the respective energies are given in Table 1, which shows that the conformer of **5** with the hydroxy hydrogen directed toward the fluorine atom (θ = 330.0°) is the most stable structure in the gas phase (this structure will be further referred to as the *global minimum*). This suggests that a F∙∙∙HO intramolecular HB is operating and governs the stability of **5**; however, different organofluorine compounds with similar orientation of the hydroxy group do not exhibit such an interaction and are highly stable [6,8]. This can be either due to other attractive interactions present in the referred conformer or prevalent repulsive interactions (e.g., between fluorine and oxygen lone pairs) in the other conformer(s). In the present study, comparison of **5** with three diastereoisomers (**6**–**8**) gives insight into the role of F∙∙∙HO intramolecular HB for the conformer stabilization, since the simple observation that the conformational energy in **5** is ca. 2.9 kcal mol^−1^ does not warrant that F∙∙∙HO intramolecular HB is the dominating, or even an operating, factor of the conformational isomerism in **5**.

**Table 1:** H–O–C–C(CF) dihedral angle (θ, in deg), relative energies, relative Lewis type energies, hyperconjugative energies, and *n*_F_→σ*_OH_ interaction energies (in kcal mol^−1^).

Diastereoisomer	θ	*E*_rel_	*E*_rel (Lewis)_	*E*_hyperconjugation_	*n*_F_→σ*_OH_

**5*** "syn-exo"*	181.0°	2.92	9.7	444.0	0.0
**5*** "syn-exo"*^ a^	330.0°	0.00	6.9	438.3	4.0
**6*** "anti-exo"*	55.5°	1.55	5.3	437.9	0.0
**6*** "anti-exo"*	182.0°	0.31	0.7	434.6	0.0
**6*** "anti-exo"*	283.2°	0.21	0.0	434.0	0.0
**7*** "syn-endo"*	72.4°	1.40	0.7	434.0	0.0
**7*** "syn-endo"*	183.7°	1.32	0.7	433.7	0.0
**8*** "anti-endo"*	53.7°	2.26	7.7	439.7	0.0
**8*** "anti-endo"*	178.0°	1.65	5.2	437.9	0.0
**8*** "anti-endo"*	288.2°	1.60	4.7	437.4	0.0

^a^*Global minimum.*

Diastereoisomers with *endo* fluorine are all above 1 kcal mol^−1^ less stable than the *global minimum*, indicating that such an orientation is less favored than the *exo* one; this behavior is independent of the orientation of the hydroxy group, since F and OH neither attract nor repel each other in the F-*endo* orientation. However, *anti-exo* conformations can be used to account for the stability of the *global minimum*, since the orientation of their fluorine atoms is the same, and thus, the intramolecular interactions with the hydroxy group are expected to explain the energetic profile. In the gas phase, two *anti-exo* conformations are marginally less stable than the *global minimum*, indicating that F∙∙∙HO intramolecular HB is operating and stabilizing. The quantum nature of this interaction can be described by the hyperconjugative interaction *n*_F_→σ*_OH_ [14], i.e., by the electron transfer from the nonbonding orbitals of fluorine to the symmetrically allowed vacant orbital σ*_OH_. Obviously, this spatial symmetry also appears for the bonding σ_OH_ orbital, giving rise to a repulsive *n*_F_/σ_OH_ interaction; the F∙∙∙HO intramolecular HB, an attractive interaction, would appear if the referred hyperconjugative interaction (plus the electrostatic nature of the F^δ−^∙∙∙^+δ^HO interaction) overrode the 4-electron/2-orbital interaction. Thus, the occurrence of the *n*_F_→σ*_OH_ interaction, which can be numerically estimated from natural bond orbital (NBO) analysis [15], is a descriptor, but not sufficient evidence, that F∙∙∙HO intramolecular HB exists. It is worth mentioning that such an interaction was calculated to be 0.9 kcal mol^−1^ in the gas phase for 2-fluorophenol (**4**), which does not exhibit F∙∙∙HO intramolecular HB [8]. Indeed, this hyperconjugative interaction was calculated at the B3LYP/aug-cc-pVDZ level to be 4.0 kcal mol^−1^ for the *global minimum* (and zero for the other structures), while the most stable *anti*-*exo* structure is only 0.2 kcal mol^−1^ less stable than the *global minimum*; clearly, there is a competition between attractive *n*_F_→σ*_OH_ and repulsive *n*_F_/σ_OH_ interactions in the *global minimum*, but its slightly higher stability compared to the *anti-exo* minimum indicates that the former interaction is prevalent.

**Figure 3:**
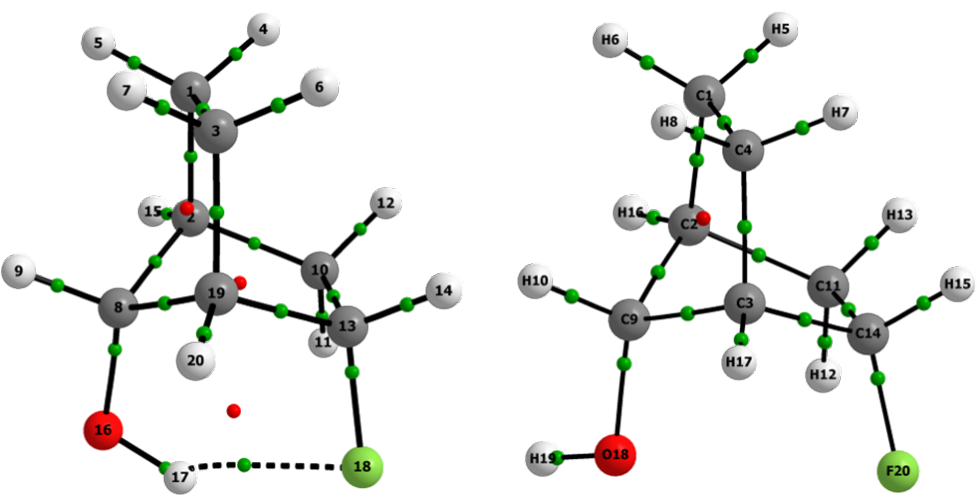
Molecular plots obtained by QTAIM for **5**. Green points represent bond critical points (BCPs) and red ones represent ring critical points (RCPs).

The F∙∙∙HO intramolecular HB in the *global minimum* was fully confirmed by Quantum Theory of AIM (QTAIM) analysis [16]. The QTAIM method is a rigorous electron density (ρ), interpretative methodology, which can define, unambiguously, atoms as they exist in molecules and the interactions between such atoms [16–18]. Even the weakest bonding interactions can be defined by the QTAIM through the so-called bond paths (BPs), that is, lines of maximum electron density linking neighboring nuclei of a molecular system in an equilibrium geometry, which, as repeatedly emphasized by Bader, is the sufficient and necessary condition for the definition of bonding between atoms [19–21]. According to Figure 3 and the QTAIM data of Table 2, the *global minimum* indeed experiences F∙∙∙HO intramolecular HB (parameters generated by using the other *syn-exo* conformer are taken as standard, because it cannot show HB), which is therefore the determining factor in its stability. Also, the stability and ionic character of the F∙∙∙HO intramolecular HB in the *global minimum* were confirmed by the low value of the ellipticity at the bond critical point (BCP, 0.04 au) and the |*V*_C_|/*G*_C_ relationship at the BCP (*V*_C_ and *G*_C_ are the kinetic and potential energy values at the F∙∙∙HO HB BCP), respectively. The |*V*_C_|/*G*_C_ parameter value is lower than 1 au (i.e., 0.973 au), and hence, the F∙∙∙HO intramolecular HB in the *global minimum* has an ionic character [22].

**Table 2:** Electronic density (ρ) and its Laplacian (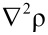) in the bond critical point (BCP) referring to the F∙∙∙HO intramolecular HB (HBCP), and the integrated properties on the H(O) atoms of the conformers of **5**.

Diastereoisomer	ρ	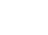	*q*(H)	*E*(H)	M_1_(H)	*V*(H)	*r*_H_	Δ*r*_H_^a^	*r*_F20_	Δ*r*_F20_^a^

**5** (θ = 181.0°)	—	—	+0.605	−0.3391	0.163	20.894	—	—	—	—
**5** (*global minimum*)	0.019	+0.075	+0.646	−0.3209	0.129	14.671	0.76	0.49	1.27	0.40

^a^Δ*r_X_* = *r_X_* − *r*^0^*_X_*, wherein *r*^0^*_X_* corresponds to the distance from the *X* nucleus (which is not involved in HB – atoms of **5** with θ = 181.0°) to the contour surface of constant 0.001 a.u., and *r_X_* corresponds to the distance from *X* to HBCP (obtained for the *global minimum*)*. r*^0^_H(O)_ = 1.25Å and *r*^0^_F_ = 1.67Å in **5** (θ = 181.0°).

In fact, according to the NBO theory, the total energy of a molecule can be split into Lewis type interactions (basically steric interactions) and electron-transfer interactions (such as hyperconjugation); this can be achieved by deleting all interactions involving antibonding and Rydberg orbitals in a molecule and then computing the energy of this hypothetical system. Accordingly, the *global minimum* was found to be the most destabilized form in terms of steric effects (possibly because of the *n*_F_/σ_OH_ contribution), but it is greatly stabilized by hyperconjugative interactions, with special emphasis on the *n*_F_→σ*_OH_ interaction (4.0 kcal mol^−1^).

**Figure 4:**
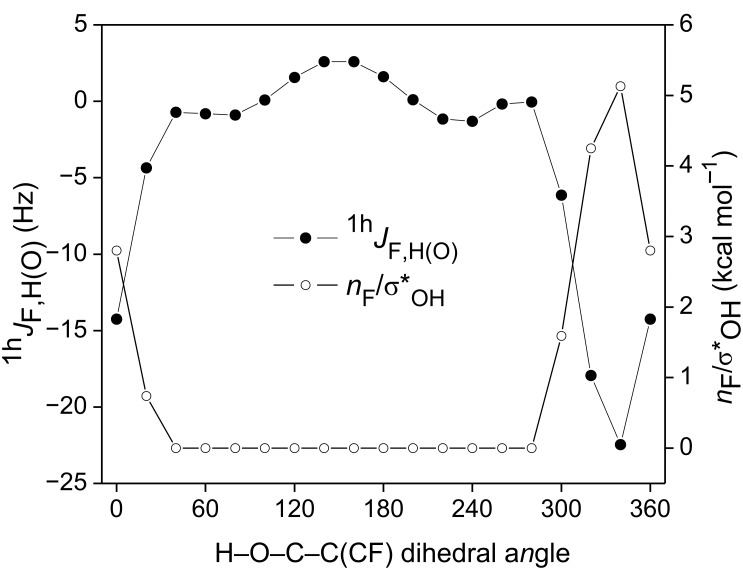
Angular dependence of ^1h^*J*_F,H(O)_ and *n_F_*→σ*_OH_ interaction in **5**.

Since the *n*_F_→σ*_OH_ interaction prevails over the *n*_F_/σ_OH_ repulsion, the F∙∙∙HO intramolecular HB can be the main transmission mechanism of a through-space F–H(O) coupling constant (^1h^*J*_F,H(O)_). This can be important for monitoring fluorine-based interactions in biological systems and material sciences. The angular dependence of ^1h^*J*_F,H(O)_ as a function of the *n_F_*→σ*_OH_ interaction was theoretically evaluated at the BHandH/EPR-III level (which has shown to perform well in estimating ^19^F-based couplings [23]), and a high correlation was found (*R*^2^ = 0.97), indicating that such an interaction, and thus the F∙∙∙HO intramolecular HB forming a six-membered ring, modulates the ^1h^*J*_F,H(O)_ SSCC (Figure 4), which is governed by the Fermi contact term. The SSCC amplitudes upon varying θ for the remaining diastereoisomers, are negligible (see Supporting Information File 1), as is the *n_F_*→σ*_OH_ interaction, as expected; on the other hand, the calculated ^1h^*J*_F,H(O)_ SSCC for the *global minimum* is significant (ca. −18 Hz, Table 3).

**Table 3:** Calculated F,H(O) SSCC for **5**–**8**, and the corresponding terms contributing to the overall *J* (FC, Fermi contact; SD, spin dipolar; PSO, paramagnetic spin-orbit; DSO, diamagnetic spin-orbit), in hertz.

Diastereoisomer	θ	FC	SD	PSO	DSO	Total *J*

**5*** "syn-exo"*	181.0°	0.67	−0.02	1.28	−1.47	0.46
**5*** "syn-exo"*^a^	330.0°	−17.68	1.41	−5.74	3.902	−18.10
**6*** "anti-exo"*	55.5°	0.63	−0.10	1.08	−1.24	0.37
**6*** "anti-exo"*	182.0°	−0.11	−0.01	0.89	−1.00	−0.24
**6*** "anti-exo"*	283.2°	0.11	−0.08	0.44	−0.48	−0.01
**7*** "syn-endo"*	72.4°	0.14	0.06	0.61	−1.13	−0.32
**7*** "syn-endo"*	183.7°	−0.36	0.05	0.01	−0.02	−0.86
**8*** "anti-endo"*	53.7°	2.92	0.01	1.05	−1.41	2.57
**8*** "anti-endo"*	178.0°	−0.58	−0.08	0.88	−0.92	−0.69
**8*** "anti-endo"*	288.2°	−0.01	0.18	0.62	−1.06	−0.28

^a^*Global minimum.*

The FC term, which dominates the ^1h^*J*_F,H(O)_ coupling in **5**, is transmitted mainly by more inner electrons than *p*-type ones, i.e., those with higher *s* % character; orbitals involved in hydrogen bonding exhibit large *s* % character [24]. The fluorine lone pairs (LP_F_) are involved in charge transfer toward the σ*_OH_ orbital, and hence, the *s* % character of these lone pairs should indicate the establishment of F∙∙∙H–O HB and, consequently, a pathway for the ^1h^*J*_F,H(O)_ coupling in **5**. While attractive interactions, such as hydrogen bonding, are expected to increase the *s* % character of the interacting lone pair, repulsive interactions are supposed to decrease such an *s* % character [24]. Accordingly, a brief comparison of the *s* % character in **5**–**8** (Table 4), obtained from the NBO analysis at the B3LYP/aug-cc-pVDZ level, indicates that a fluorine lone pair (LP_F_(1)) is preponderantly involved in repulsive interactions (such as *n*_F_/σ_OH_), while LP_F_(3) participates in interactions that are preponderantly attractive; summed up, the larger *s* % character of the *n*_F_ lone pairs in the *global minimum* compared to the diastereoisomers that are not capable of exhibiting HB, indicates an overall (slight) attractive interaction between F and OH, in agreement with the small energy difference between the *global minimum* and the second-most stable structure (*anti-exo*, θ = 283.2°).

**Table 4:** The *s* % character of LP_F_ in **5**–**8**.

Diastereoisomer	θ	LP_F_(1)	LP_F_(2)	LP_F_(3)

**5*** "syn-exo"*	181.0°	71.85%	0.36%	0.03%
**5*** "syn-exo"*^a^	330.0°	71.81%	0.00%	1.01%
**6*** "anti-exo"*	55.5°	72.38%	0.12%	0.02%
**6*** "anti-exo"*	182.0°	72.41%	0.11%	0.01%
**6*** "anti-exo"*	283.2°	72.43%	0.13%	0.01%
**7*** "syn-endo"*	72.4°	72.19%	0.05%	0.02%
**7*** "syn-endo"*	183.7°	72.17%	0.04%	0.02%
**8*** "anti-endo"*	53.7°	72.21%	0.05%	0.01%
**8*** "anti-endo"*	178.0°	72.13%	0.06%	0.01%
**8*** "anti-endo"*	288.2°	72.17%	0.06%	0.00%

^a^*Global minimum.*

## Conclusion

In summary, there is a competition between *n*_F_→σ*_OH_ and *n*_F_/σ_OH_ interactions as driving forces of the conformational isomerism of **5**, but the former is slightly dominant, modulates the ^1h^*J*_F,OH_ SSCC in this aliphatic organofluorine compound, and is the main factor responsible for the large value of ^1h^*J*_F,OH_ in the *global minimum*.

## Supporting Information

Supporting Information contains experimental procedures for newly synthesized compounds and NMR spectra.

File 1 Angular dependences of SSCCs and energies in **5**–**8**.
