# Supplementary material for: Conformational analysis, stereoelectronic interactions and NMR properties of 2-fluorobicyclo[2.2.1]heptan-7-ols
Source: Beilstein J Org Chem. 2012 Aug 2;8:1227–32. doi: 10.3762/bjoc.8.137 (PMC3458742; doi:10.3762/bjoc.8.137)
Supplement: File 1 — Angular dependences of SSCCs and energies in 5–8. [file Beilstein_J_Org_Chem-08-1227-s001.pdf]

Supporting Information

for

Conformational analysis, stereoelectronic interactions and NMR properties of 2-fluorobicyclo[2.2.1]heptan-7-ols

Fátima M. P. de Rezende[1], Marilua A. Moreira[1], Rodrigo A. Cormanich[2] and Matheus P. Freitas[*1]

Address: [1]Department of Chemistry, Federal University of Lavras, P.O. Box 3037, 37200-000, Lavras, MG, Brazil and [2]Chemistry Institute, State University of Campinas, P.O. Box 6154, 13083-970, Campinas, SP, Brazil

Email: Matheus P. Freitas - matheus@dqi.ufla.br

[*] Corresponding author

Angular dependences of SSCCs and energies in 5–8

Figure S1: Angular dependence of $J_{F,H(O)}$ and its components FC (*Fermi Contact*), SD (*Spin Dipolar*), PSO (*Paramagnetic Spin-Orbit*) and DSO (*Diamagnetic Spin-Orbit*) in **5**.

Figure S2: Angular dependence of $J_{F,H(O)}$ and its components FC (*Fermi Contact*), SD (*Spin Dipolar*), PSO (*Paramagnetic Spin-Orbit*) and DSO (*Diamagnetic Spin-Orbit*) in **6**.

Figure S3: Angular dependence of $J_{F,H(O)}$ and its components FC (*Fermi Contact*), SD (*Spin Dipolar*), PSO (*Paramagnetic Spin-Orbit*) and DSO (*Diamagnetic Spin-Orbit*) in **7**.

Figure S4: Angular dependence of $J_{F,H(O)}$ and its components FC (*Fermi Contact*), SD (*Spin Dipolar*), PSO (*Paramagnetic Spin-Orbit*) and DSO (*Diamagnetic Spin-Orbit*) in **8**.

Figure S5: Angular dependence of total hyperconjugation in **5**–**8**.

Figure S6: Angular dependence of Lewis-type energies in **5**–**8**.

Figure S7: Angular dependence of $^{1}J_{F,H(O)}$ and $n_F \rightarrow \sigma^*_{OH}$ in **5**.

Figure S8: Angular dependence of $^{1}J_{F,H(O)}$ and $n_F \rightarrow \sigma^*_{OH}$ in **6**.

Figure S9: Angular dependence of $^{1}J_{F,H(O)}$ and $n_F \rightarrow \sigma^*_{OH}$ in **7**.

Figure S10: Angular dependence of $^{1}J_{F,H(O)}$ and $n_F \rightarrow \sigma^*_{OH}$ in **8**.

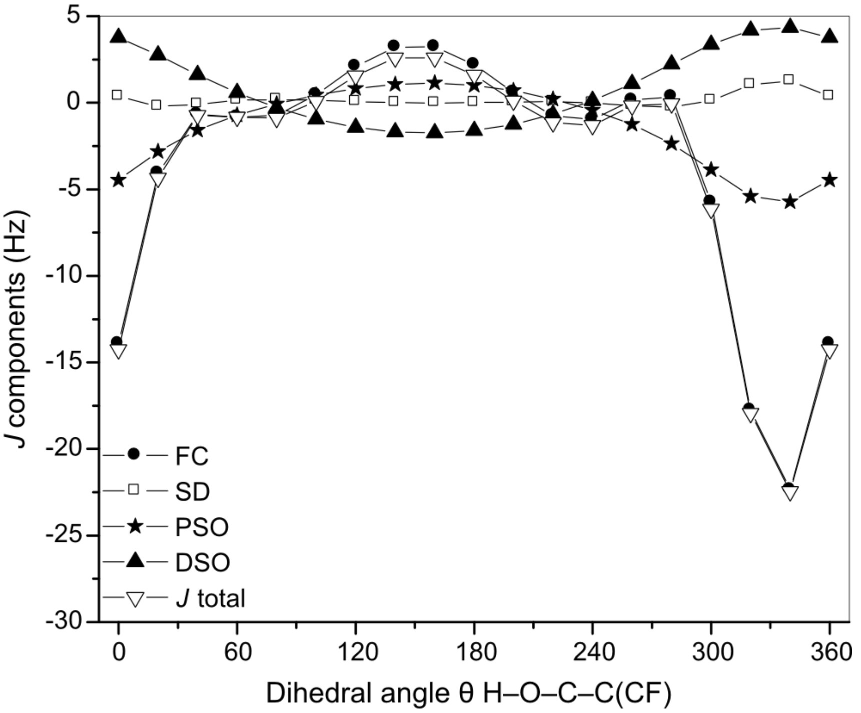

Figure S1: Angular dependence of $J_{F,H(O)}$ and its components FC (*Fermi Contact*), SD (*Spin Dipolar*), PSO (*Paramagnetic Spin-Orbit*) and DSO (*Diamagnetic Spin-Orbit*) in **5**.

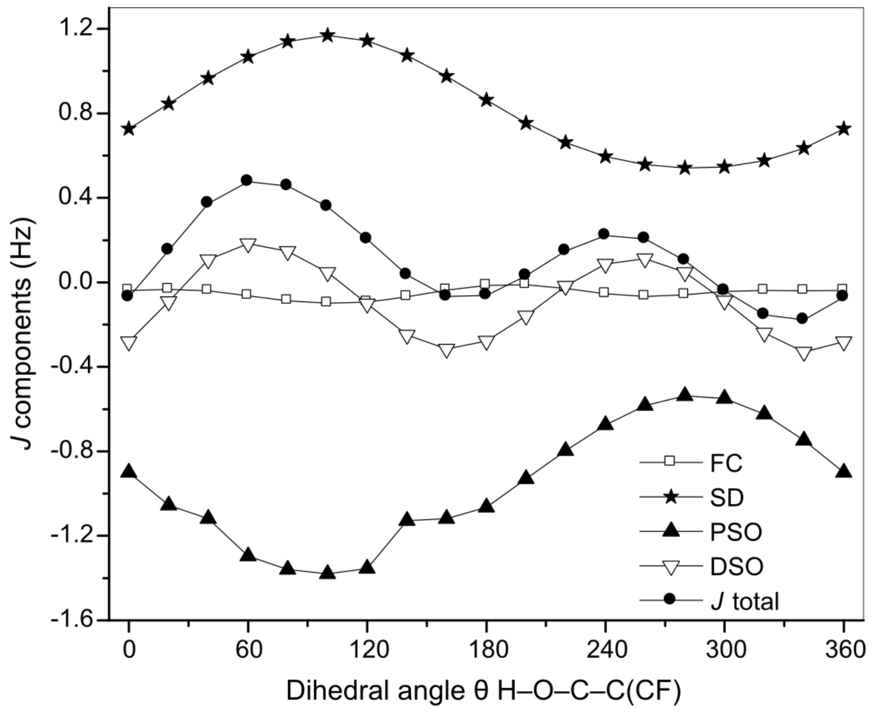

Figure S2: Angular dependence of $J_{F,H(O)}$ and its components FC (*Fermi Contact*), SD (*Spin Dipolar*), PSO (*Paramagnetic Spin-Orbit*) and DSO (*Diamagnetic Spin-Orbit*) in **6**.

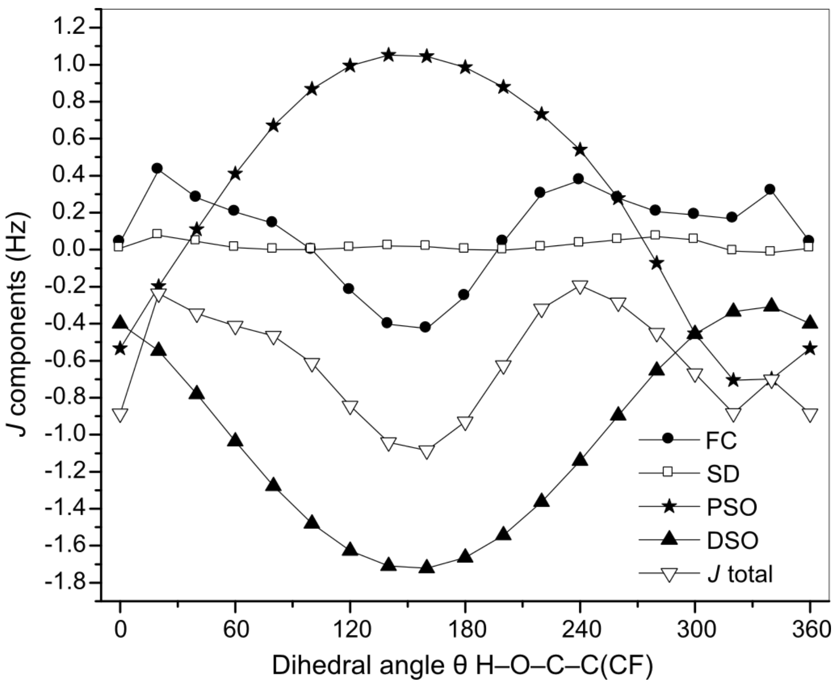

Figure S3: Angular dependence of $J_{F,H(O)}$ and its components FC (*Fermi Contact*), SD (*Spin Dipolar*), PSO (*Paramagnetic Spin-Orbit*) and DSO (*Diamagnetic Spin-Orbit*) in **7**.

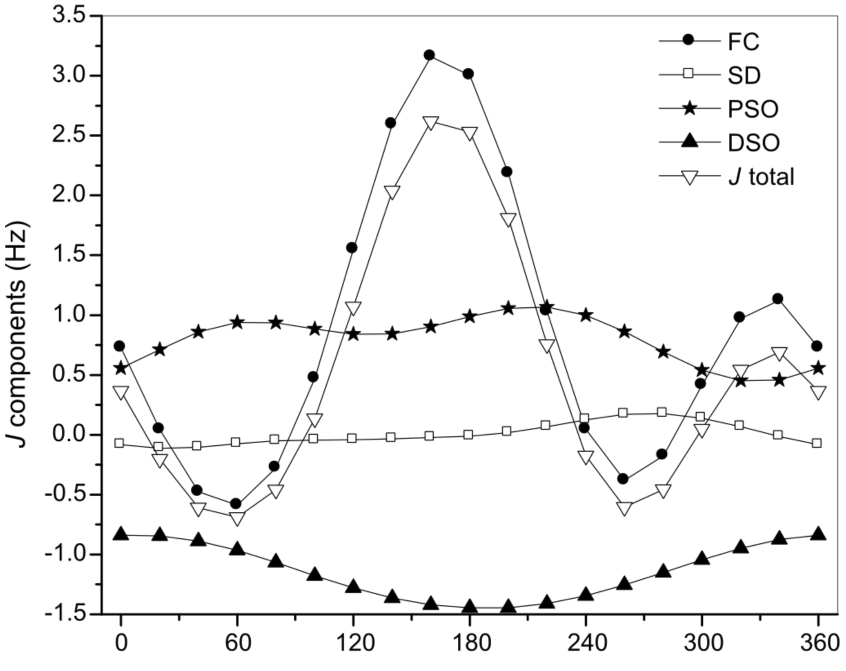

Figure S4: Angular dependence of $J_{F,H(O)}$ and its components FC (*Fermi Contact*), SD (*Spin Dipolar*), PSO (*Paramagnetic Spin-Orbit*) and DSO (*Diamagnetic Spin-Orbit*) in **8**.

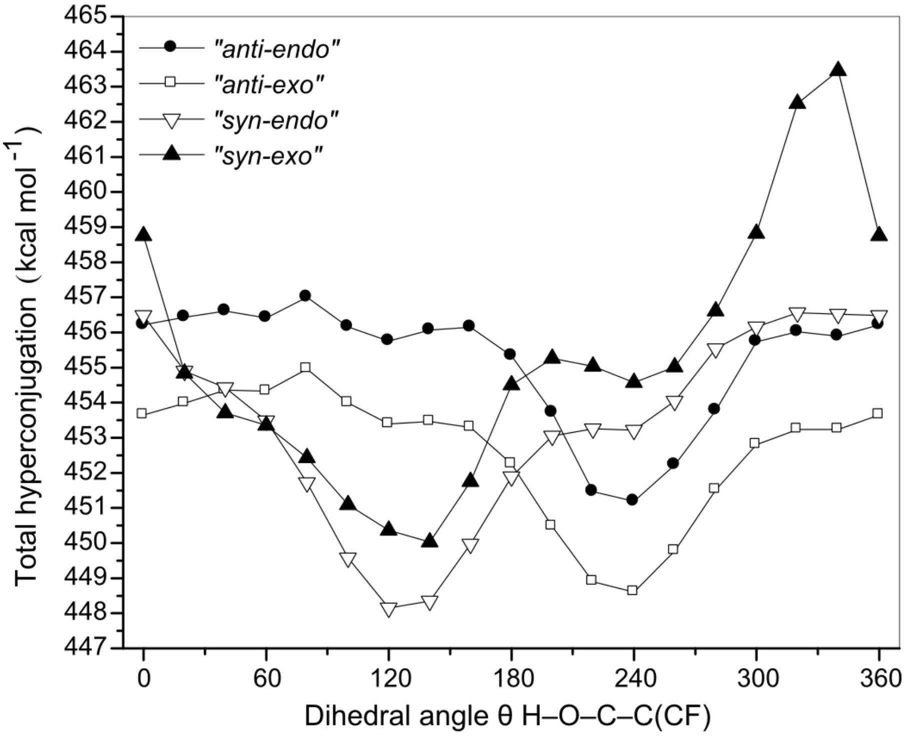

Figure S5: Angular dependence of total hyperconjugation in **5**–**8**.

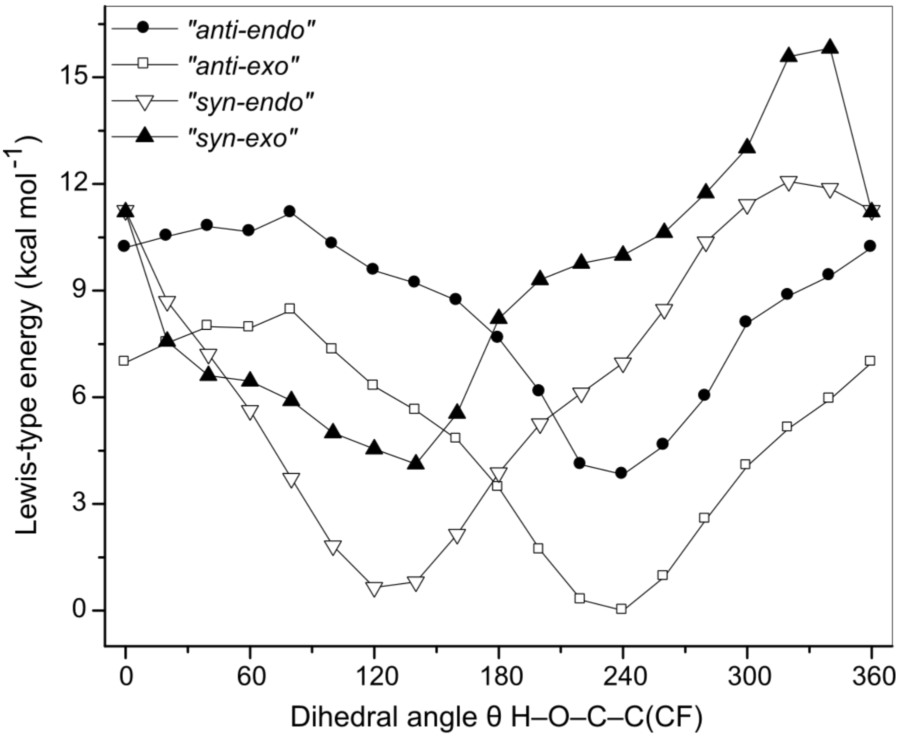

Figure S6: Angular dependence of Lewis-type energies in **5**–**8**.

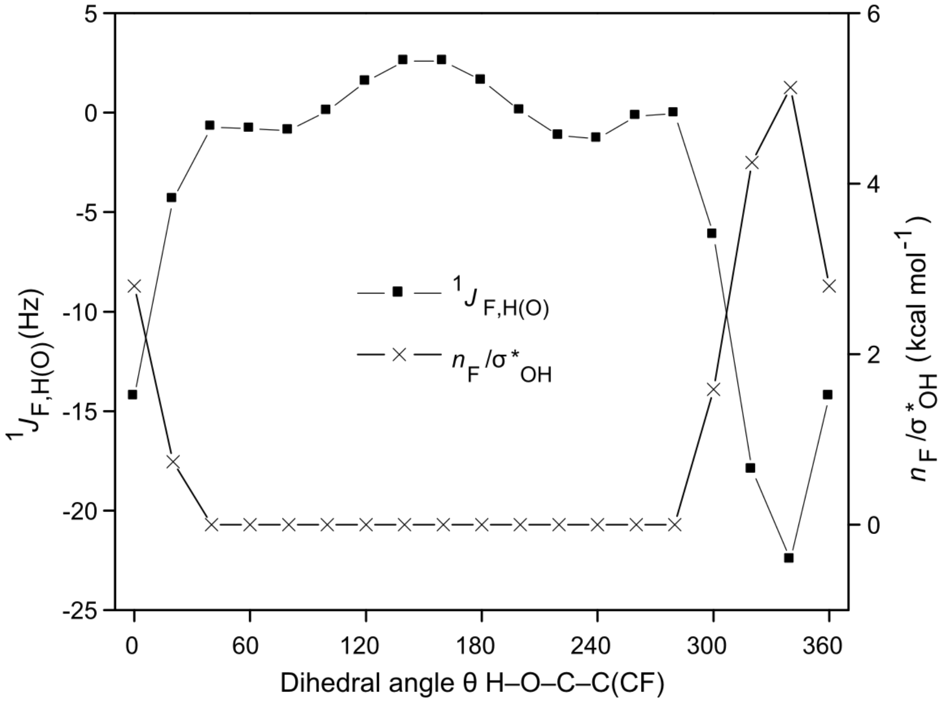

Figure S7: Angular dependence of $^1J_{F,H(O)}$ and $n_F{\rightarrow}\sigma^*_{OH}$ in **5**.

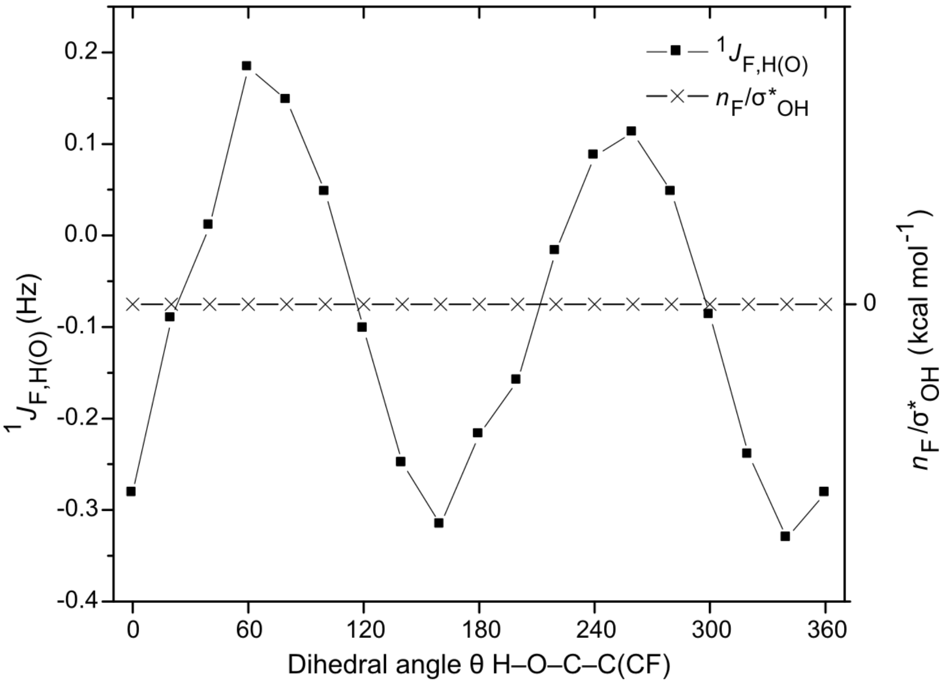

Figure S8: Angular dependence of $^1J_{F,H(O)}$ and $n_F{\rightarrow}\sigma^*_{OH}$ in **6**.

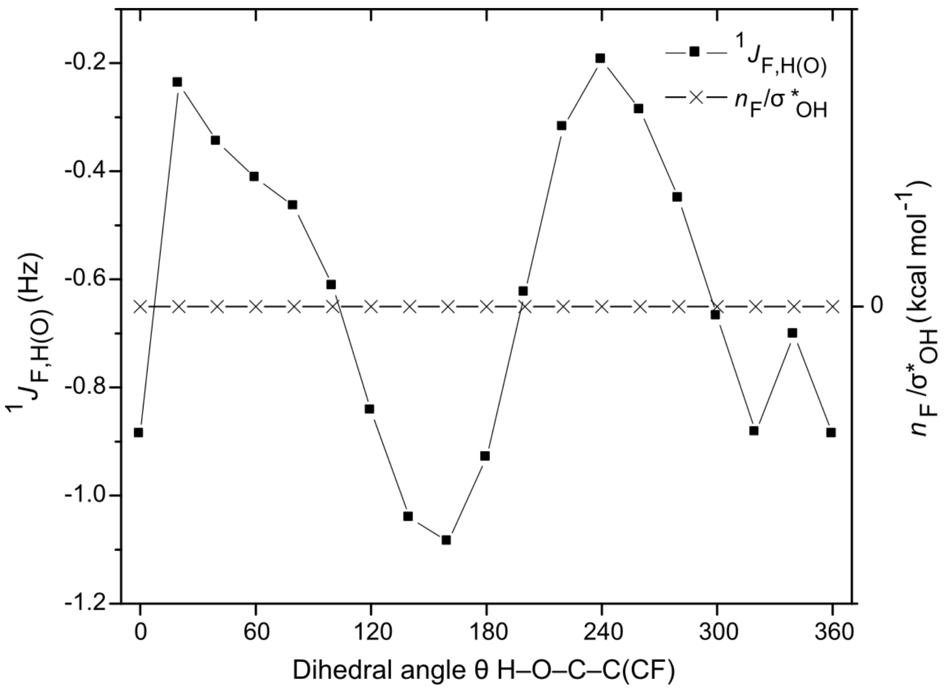

Figure S9: Angular dependence of ${}^{1}J_{F,H(O)}$ and $n_F \rightarrow \sigma^*_{OH}$ in **7**.

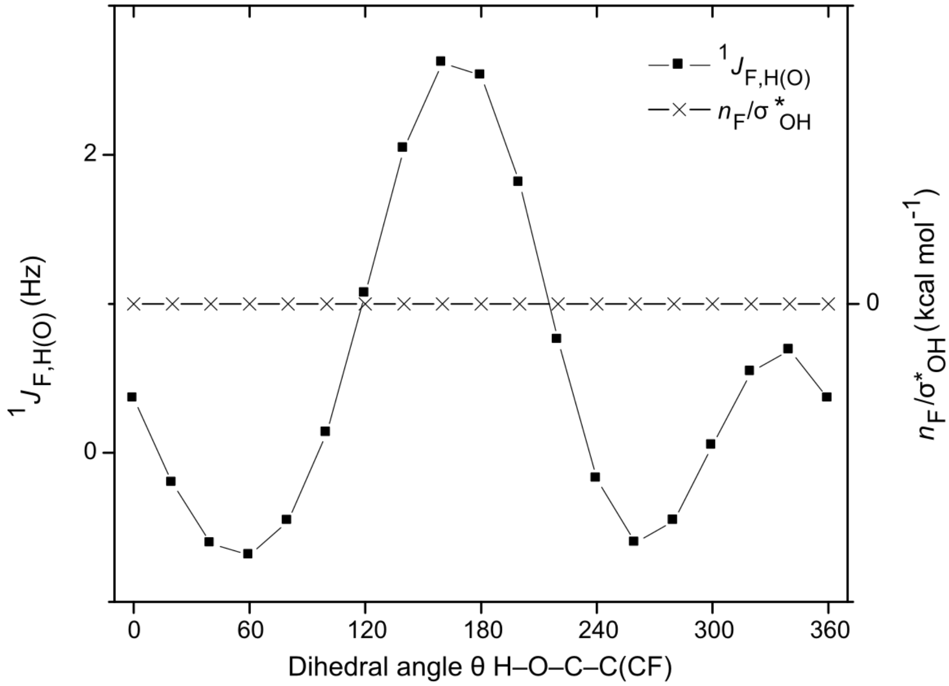

Figure S10: Angular dependence of ${}^{1}J_{F,H(O)}$ and $n_F \rightarrow \sigma^*_{OH}$ in **8**.
